# Use of tetraethylammonium (TEA) and Tris loading for blocking TRPM7 channels in intact cells

**DOI:** 10.3389/fphar.2024.1341799

**Published:** 2024-04-10

**Authors:** Katherine G. Holderby, J. Ashot Kozak

**Affiliations:** ^1^ Undergraduate Program in Physiology and Neuroscience, Dayton, OH, United States; ^2^ Department of Neuroscience, Cell Biology and Physiology, Boonshoft School of Medicine and College of Science and Mathematics, Wright State University, Dayton, OH, United States

**Keywords:** lymphocyte, channel-kinase, HEK293, tetramethylammonium ion, tetraethylammonium ion, Tris hydrochloride, Jurkat T cell, magnesium

## Abstract

Tetraethylammonium (TEA), a quaternary ammonium compound, is a well-known blocker of potassium channels belonging to various subfamilies, such as K_V_1-3, K_Ca_1, 2 and prokaryotic KcsA. In many cases, TEA acts from the extracellular side by open pore blockade. TEA can also block transient receptor potential (TRP) cation channels, such as TRPM7, in a voltage-dependent manner. In human T lymphocytes, intracellular (cytosolic) TEA and its analog TMA (tetramethylammonium) inhibit TRPM7 channel currents in the outward but not inward direction. By contrast, intracellular Mg^2+^, protons and polyamines inhibit both outward and inward current components equally. Likewise, the majority of available pharmacological tools inhibit TRPM7 channels in a voltage-independent manner. Since TRPM7 is a steeply outwardly rectifying conductance, voltage-dependent blockers can be useful for studying the cellular functions of this channel. TRPM7 protein is endogenously expressed in diverse cell lines, including HEK, HeLa, CHO, RBL and Jurkat. Using patch-clamp electrophysiology, we found that incubating HEK293 and Jurkat T cells overnight in the presence of 20 mM TEA-Cl, resulted in the nearly complete blockade of whole-cell TRPM7 outward current, measured at break-in. By contrast, the inward current was unchanged in TEA-loaded cells. The blockade was fully reversible after washout of intracellular solution in whole-cell but not in perforated-patch recording configurations. Overnight incubation with 20 mM TMA-Cl resulted in a more modest blockade of the outward TRPM7 current. Internal 129 mM TMA and TEA eliminated most of the outward current. TEA uptake in transfected HEK293 cells led to blockade of recombinant murine TRPM7 and the Mg^2+^ and pH insensitive Ser1107Arg variant. Unexpectedly, Tris-HCl, a widely used pH buffer, could similarly be loaded into Jurkat and HEK cells, and preferentially blocked outward TRPM7 currents. 20 mM and 129 mM Tris in the internal solution blocked TRPM7 current in outward but not inward direction. Voltage-dependent channel blockade by TEA, TMA and Tris loading will be useful for studying the properties and functions of TRPM7-mediated ion transport in intact cells.

## Materials and methods

### Cell culture

Human embryonic kidney HEK293 and Jurkat Clone E61 leukemic T cells were purchased from ATCC (Manassas, VA) and maintained in RPMI 1640 (Gibco, Thermo-Fisher Scientific, Waltham, MA) supplemented with 10% fetal bovine serum (FBS) (Biowest USA, Brandenton, FL) and penicillin/streptomycin (Gibco) in a CO_2_ incubator at 37°C (Forma Scientific, Marietta, OH) (Chokshi et al., 2012b). Jurkat T cells were passaged 2 or 3 times a week by diluting the cell suspension in fresh culture medium. HEK293 cells were passaged twice a week using trypsin-EDTA (Life Technologies, Grand Island, NY) and Cellstripper reagent (Mediatech, Manassas, VA). The normal RPMI medium contains 0.42 mM [Ca^2+^] and 0.4 mM [Mg^2+^] (Mellott et al., 2020). For preparing the low magnesium medium, custom-made divalent metal free (DVF) RPMI (Gibco) was supplemented with 10% FBS, treated with Chelex 100 sodium resin beads (Sigma-Aldrich, St. Louis, MO) and passed through a sterile 0.22 µm polyethersulfone filter (Thermo-Fisher). 0.42 mM CaCl_2_, 10 µM HEDTA, 10 µM MgCl_2_ were added to Chelex-treated DVF RPMI, yielding approximately 8 µM free Mg^2+^. In a few experiments, 40 µM HEDTA was used with 0.42 mM CaCl_2_ and 10 µM MgCl_2_ (Mellott et al., 2020). The high potassium medium (see Figure 5) was prepared by adding 25 mM KCl to RPMI medium, yielding 30.3 mM final K^+^ concentration.

### Heterologous expression of murine wildtype TRPM7 and S1107R variant

HEK293 cells were chemically transfected with pEGFP-mTRPM7 and pEGFP-mTRPM7 S1107R plasmids using polyethyleneimine (PEI) (Polysciences, Warrington, PA) or LT1 (Mirus Bio, Madison, WI) by mixing with DNA and Dulbecco’s phosphate-buffered saline (DPBS) and adding to cells in 6-well polystyrene plates (Corning Incorporated, Kennebunk, ME) (Zhelay et al., 2018). Plasmid DNA was purified using PureYield plasmid midiprep kit (Promega, Madison, WI).

### Patch clamp electrophysiology

Endogenous and over-expressed TRPM7 channel currents were recorded in whole-cell and perforated patch configurations in HEK293 and Jurkat T cells as previously described in detail (Kozak et al., 2002; Chokshi et al., 2012b; Mellott et al., 2020). Briefly, on the day of experiment cells were transferred to a glass-bottom plexiglass recording chamber grounded with a silver-silver chloride electrode connected to EPC10 amplifier headstage (HEKA Elektronik, Lambrecht, Germany). Patch pipettes were manufactured from borosilicate glass capillaries (Harvard Apparatus, Holliston, MA) using a horizontal P-1000 puller (Sutter Instrument, Novato, CA). The pipette tips were fire-polished on a microforge (MF-830, Narishige, Tokyo, Japan), yielding resistances of ∼2–3 MOhm. TRPM7 currents were recorded by applying −100 to 100 mV command voltage ramps every 1.5 s (Chokshi et al., 2012b). The voltage was held at 0 mV between ramps. The standard “0.4 Ca/0.4 Mg” bath (external) solution contained (in mM): 0.4 CaCl_2_, 0.4 MgCl_2_, 140 sodium aspartate, 4.5 KCl, 3 CsCl, 10 HEPES sodium, pH 7.3. In some experiments the external solution contained 2 mM CaCl_2_ and no Mg^2+^ (see Figure 6). “DVF” external solution contained (in mM) 6 mM HEDTA, 140 aspartic acid, 10 HEPES acid, pH 7.3 with CsOH. The whole-cell internal solution contained (in mM) 112 glutamic acid, 10 HEDTA, 8 NaCl, 10 HEPES acid, pH 7.3 with CsOH. In experiments with TEA-Cl and Tris-HCl addition, the internal solution contained (in mM) 100 glutamic acid, 10 HEDTA, 8 NaCl, 10 HEPES acid, 133 CsOH, pH 7.3. For 129 mM internal TEA, TMA and Tris-HCl experiments the pipette solution contained: 129 mM TEA, TMA or Tris-HCl, 5 mM CsF, 1 mM EGTA, 10 mM HEPES, pH 7.3. Osmolalities of all solutions were measured with a freezing point osmometer (Osmette III, Precision Systems, Natick, MA) and adjusted with D-mannitol as needed. Aliquots of 1 M TEA-Cl, 1 M TMA-Cl, 1 M Tris-HCl prepared in deionized water were added to cell cultures at specified final concentrations for 1–3 days. Perforated-patch recordings were performed as previously described (Kozak et al., 2005; Zhelay et al., 2018; Mellott et al., 2020). A frozen aliquot of amphotericin B (Sigma) stock solution in DMSO (6 mg/10 µL) was thawed and diluted in the recording solution which contained 50 mM Cs_2_SO_4_, 50 mM CsCl, 7 mM MgCl_2_, 1 mM CaCl_2_, 10 mM HEPES, pH 7.3. Pipette tips were briefly immersed in antibiotic-free recording solution before backfilling with antibiotic-containing solution. After gigaohm seal formation, successful perforation was assessed as a gradual reduction in access resistance occurring over ∼10–20 min. Recordings were discarded if the access resistance exceeded 20 MOhm or a spontaneous break-in occurred. All patch clamp recordings were performed at room temperature in the absence of TEA, TMA and Tris in the bath solution. In HEK293 cells transfected with TRPM7 constructs, the recordings were performed 2–3 days after transfection, and successfully transfected cells were identified by GFP fluorescence on an inverted Nikon microscope equipped with a fluorescent illumination system (89 North, Williston, VT).

### Data analysis

In order to quantify the extent of block in TEA and Tris loaded cells in the Ca^2+^-containing bath solution, TRPM7 current magnitude measured at break-in (I_0_) was divided by maximum current magnitude after washout (I_max_) measured at 98.67 mV. For block of the monovalent TRPM7 current recorded in DVF, the break-in current magnitude at 98.67 mV was divided by current magnitude at −100 mV (R = I_0_ (+98.67)/I_0_ (−100), followed by division by the ratio of maximum current magnitudes (I_max_) at these voltages (r = I_max_ (+98.67)/I_max_ (−100). R/r ratio accounts for differences in the degree of monovalent TRPM7 current preactivation (basal current magnitude) due to cellular Mg^2+^ depletion (Mellott et al., 2020). In some recordings the washout trace was normalized to the amplitude of the break-in trace measured at −100 mV as specified in figure legends. Two sample Student’s t-test was used for statistical comparisons of means. Patchmaster (HEKA), Microsoft Excel and Origin (v. 2017, 9.0, Originlab, Northampton, MA) were used for data analysis and graphing.

### Chemicals

DPBS was purchased from HyClone (Logan, UT). Aspartic acid, CsOH, Cs_2_SO_4_, CsCl and D-mannitol were from Sigma. Tetraethylammonium chloride (TEA-Cl), 1.0 M CaCl_2_ and 1.0 M MgCl_2_ were from Fluka Honeywell (Muskegon, MI). Glutamic acid, HEDTA, HEPES sodium, tetramethylammonium chloride (TMA-Cl) and NaCl were from Thermo Fisher. HEPES acid and sodium aspartate were from Alfa Aesar (Ward Hill, MA). KCl was from Fisher Scientific. Tris(hydroxymethyl)aminomethane hydrochloride (Tris-HCl) was from Promega (Madison, WI).

## Introduction

TRPM7 (transient receptor potential melastatin 7) channels belong to the superfamily of TRP cation channels, and are highly expressed in cells of the immune system: lymphocytes, macrophages, mast cells and neutrophils (Nadolni and Zierler, 2018; Hoeger and Zierler, 2022; Liang et al., 2022; Alavi et al., 2024). TRPM7 is expressed in widely used human cell lines such as Jurkat, HEK293, HeLa, K562, MCF7 (Numata et al., 2007; Dhennin-Duthille et al., 2011; Chokshi et al., 2012b; Takahashi et al., 2018). This protein is unique in that its C-terminus includes a functional serine/threonine protein kinase domain (Yamaguchi et al., 2001; Ryazanova et al., 2004; Matsushita et al., 2005; Visser et al., 2014). TRPM7 channels are sensitive to cytosolic Mg^2+^, pH and other divalent metal cations (Kozak et al., 2002; Kozak and Cahalan, 2003; Chokshi et al., 2012b; c). Intracellular magnesium nucleotides such as MgATP and MgGTP were also reported to inhibit TRPM7 channels (Demeuse et al., 2006).

Pharmacological tools often used in TRPM7 research include channel inhibitors SKF-96365, 2-APB, spermine, philanthotoxin, NS8593, FTY-720, quinine, salicylate, lanthanides and others (Kozak et al., 2002; Kerschbaum et al., 2003; Gwanyanya et al., 2004; Wykes et al., 2007; Chokshi et al., 2012a; Chubanov et al., 2012; Qin et al., 2013; Mellott et al., 2020; Chokshi et al., 2021). Several TRPM7 channel blockers (2-APB, naproxen, ibuprofen, salicylate, acetylsalicylate) inhibit TRPM7 channels through a cytosolic acidification mechanism (Chokshi et al., 2012a; Chokshi et al., 2021). Unfortunately, TRPM7 inhibitors are not specific and target other channels and receptors (Cheng et al., 2022). The majority inhibit TRPM7 in a voltage-independent manner. By contrast, external polyamines (spermine, spermidine, putrescine, philanthotoxin), tetraethylammonium (TEA), tetramethylammonium (TMA) and La^3+^ are voltage-dependent blockers (Kozak et al., 2005). External La^3+^, at concentrations not exceeding 1 mM, blocks only the inward TRPM7 current (Kozak and Cahalan, 2001; Mellott et al., 2020). Polyamines block the inward monovalent TRPM7 current at micromolar concentrations (Kozak et al., 2002; Kerschbaum et al., 2003), whereas intracellular TEA and TMA block the outward monovalent current at millimolar concentrations (Kozak et al., 2005). At concentrations of 200 µM and higher, external polyamines also block the outward TRPM7 current (Kerschbaum et al., 2003).

The tools available for specifically eliminating the outward TRPM7 current have been limited, since TEA and TMA blockade occurs when added to the internal recording solution. In the present study, we report that in intact HEK293 and Jurkat T cells, TEA, TMA blocked outward TRPM7 current by overnight incubation with these compounds. Additionally, we discovered that intracellular Tris-HCl (tris(hydroxymethyl)aminomethane), is a voltage-dependent blocker of TRPM7 channels that can enter HEK293 and Jurkat cytosol from the outside. TEA was an effective blocker of recombinant murine TRPM7 and its constitutively active variant S1107R (Zhelay et al., 2018). Thus, TEA, TMA, Tris accumulate in cell cytosol and inhibit the outward TRPM7 current in a concentration-dependent manner, without affecting the inward current. We propose that cellular uptake of these compounds can be useful for dissecting the functions of TRPM7 current components.

## Results

### Blockade of native TRPM7 channels in cells loaded with TEA and TMA

Previously, we showed that 20 mM TEA or 20 mM TMA in the internal solution blocks whole-cell monovalent TRPM7 current in a voltage-dependent manner. Both TEA, and to a lesser degree TMA, reduced the magnitude of the outward but not inward current in human T lymphocytes (Kozak et al., 2005). In order to evaluate if these compounds could also be useful for blocking outward TRPM7 currents in intact cells, we incubated Jurkat T cells overnight in Mg^2+^- deficient RPMI medium, in the presence of TEA. Maintaining Jurkat T cells in 8 µM [Mg^2+^]/0.42 mM [Ca^2+^] medium for 1–3 days results in cytosolic Mg^2+^ depletion and almost complete activation of TRPM7 current (Mellott et al., 2020). In cells maintained under normal Mg^2+^ conditions ([Mg](RPMI) = 0.4 mM), the majority of TRPM7 channels are inhibited, and full current activation requires 5–10 min of recording with Mg^2+^-free internal solution (Chokshi et al., 2012b; Mellott et al., 2020).

**FIGURE 1:**
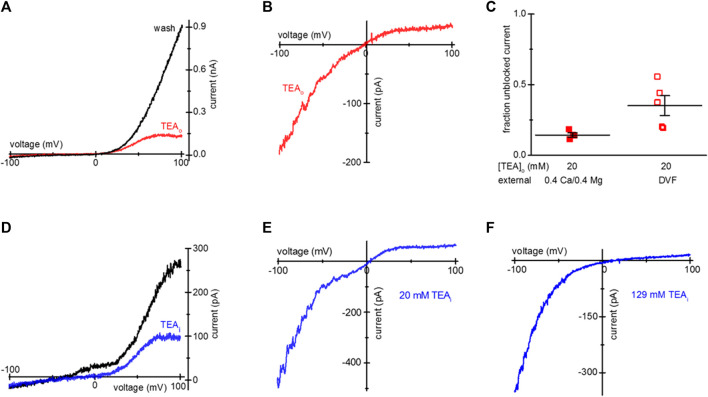
Blockade of TRPM7 current in TEA-loaded Mg^2+^- depleted Jurkat T cells. **(A)** TRPM7 I-V relations at break-in (red trace) and after 20 mM TEA-Cl washout (black) recorded in 0.4 Ca/0.4 Mg external solution. **(B)** Monovalent TRPM7 current I-V in a 20 mM TEA-Cl loaded cell at break-in. **(C)** Summary of TRPM7 current blockade by uptake of 20 mM TEAo, measured in 0.4 Ca/0.4 Mg (filled squares) and DVF (empty squares) external solutions. **(D)** Blockade of TRPM7 current by 20 mM TEA added to the internal solution. I-V relations at break-in (black trace) and after blockade (blue) with 20 mM TEA. **(E,F)** Blockade of monovalent TRPM7 current by internal 20 mM and 129 mM TEA. Jurkat T cells were incubated in low Mg^2+^ RPMI **(A–D)** supplemented with TEA-Cl **(A–C)** for 1–2 days. In **(D–F)** the cells were not exposed to external TEA.

Activation of TRPM7 current in intact cells by prior Mg^2+^ depletion, allows the study of TEA blockade separately from Mg^2+^ inhibition. Additionally, it separates internal Mg^2+^ washout from TEA washout. Figure 1A shows whole-cell current-voltage (I-V) relations obtained after overnight incubation with 20 mM TEA, at break-in (red trace) and after several minutes of recording (black). The break-in (t = 0) trace represents the current present in an intact cell in external 0.4 Ca/0.4 Mg, before cytosol exchange with the pipette solution. The I-V was modified by a preferential reduction of current at positive membrane potentials. TEA blockade was fully reversible after exchange with TEA-free internal solution (Figure 1A).

Upon removal of external divalent cations (e.g., Ca^2+^ and Mg^2+^), TRPM7 current becomes semi-linear due to removal of tonic inward current blockade (Kozak et al., 2002). Thus, it is possible to study the effect of the blockers in the full voltage range by recording TRPM7 monovalent currents. We, therefore, recorded monovalent TRPM7 currents in external DVF. As expected, monovalent current was blocked in the outward direction, giving rise to inward rectification (Figure 1B). The extent of 20 mM TEA block in 0.4 Ca/0.4 Mg and DVF is summarized in Figure 1C. For comparison, inclusion of 20 mM TEA in the pipette resulted in blockade of outward TRPM7 currents both in 0.4 Ca/0.4 Mg and in DVF (Figures 1D, E). 129 mM internal TEA eliminated almost all monovalent outward current without affecting the inward current (Figure 1F). These experiments demonstrated that after prolonged incubation, TEA enters intact Jurkat cells, blocks TRPM7 channels from the cytosolic side, and the blockade is reversible.

**FIGURE 2:**
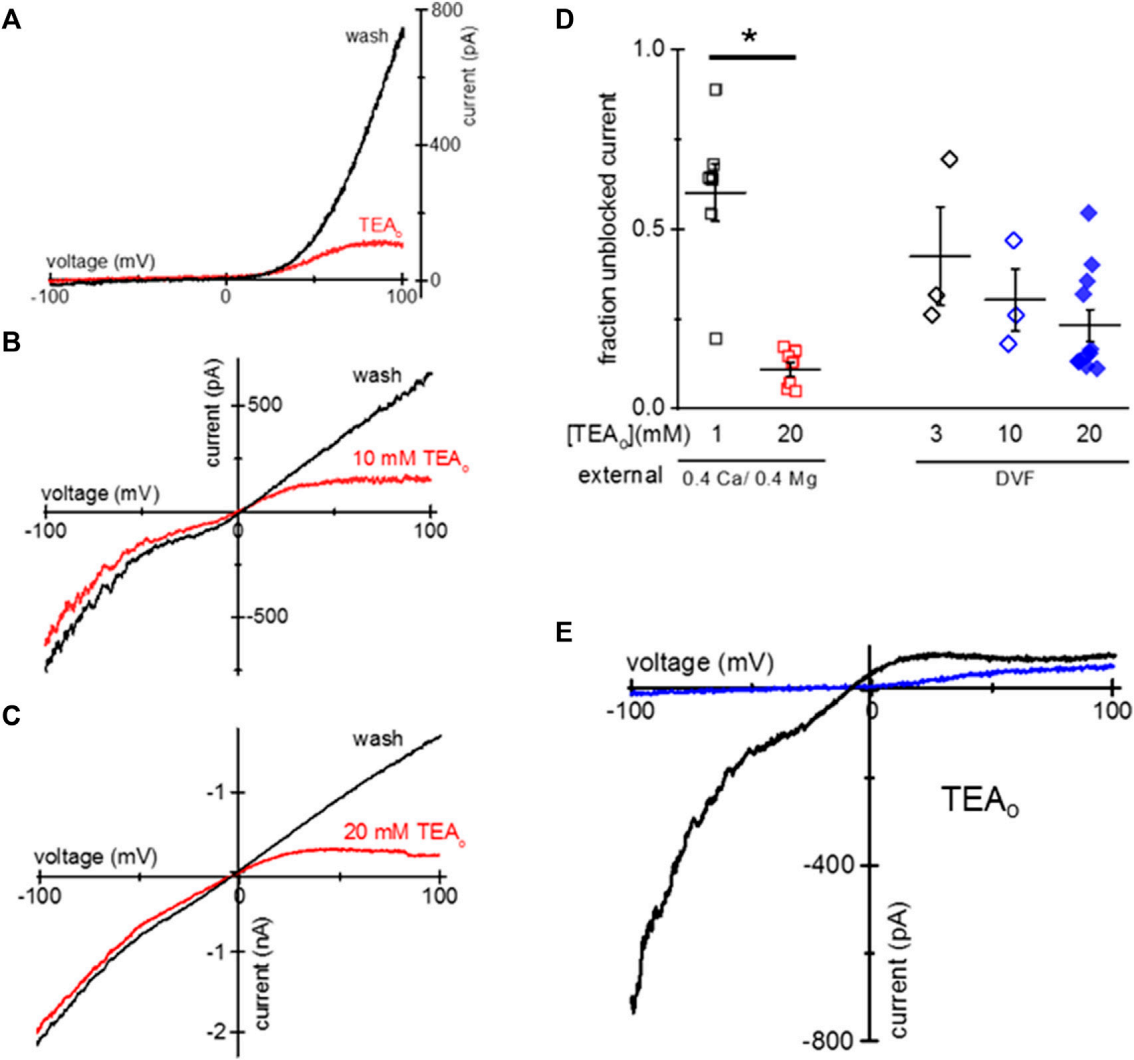
Blockade of TRPM7 current in TEA-loaded Mg^2+^- depleted HEK293 cells. **(A)** TRPM7 I-V relations at break-in (red trace) and after 20 mM TEA washout (black) in 0.4 Ca/0.4 Mg solution. **(B,C)** Monovalent TRPM7 I-V in 10 mM and 20 mM TEA loaded cells at break-in and after washout. **(D)** Summary of TRPM7 current blockade by uptake of indicated concentrations of TEA_o_ for 0.4 Ca/0.4 Mg and DVF external solutions. **(E)** Perforated patch recording of TRPM7 current in 0.4 mM Ca/0.4 mM Mg (blue) and DVF (black) in a cell loaded with 20 mM TEA. No washout of TEA block was observed during perforated-patch recording. HEK cells were incubated in low Mg^2+^ RPMI supplemented with TEA for 1–2 days **(A–E)**. Asterisk denotes Student’s two sample test *p* < 0.05.

HEK293 cells endogenously express TRPM7 channels, and are commonly used for heterologous expression of TRPM7 (Chokshi et al., 2012c; Zhelay et al., 2018). We, therefore, tested if TEA uptake can occur in HEK293 cells. We incubated HEK293 cells overnight in low Mg^2+^ RPMI and 1–20 mM TEA. Similar to Jurkat T cells, this treatment resulted in cytosolic Mg^2+^ depletion and activation of TRPM7 channels concomitant with TEA uptake and voltage-dependent blockade. Figures 2A–C show TEA blockade of endogenous TRPM7 channels in HEK293 cells. Figure 2A shows I-V relations obtained in 0.4 Ca/0.4 Mg at break-in and after TEA washout. Figures 2B, C show monovalent TRPM7 current blocked in cells incubated with 10 mM and 20 mM TEA, respectively. The blockade occurred only in the outward direction. The summary of TRPM7 current blockade by various concentrations of TEA in HEK293 cells is presented in Figure 2D.

We performed similar experiments using amphotericin perforated-patch recording, which prevents the exchange of divalent ions between the cytosol and the pipette solution (Rae et al., 1991; Mellott et al., 2020). Figure 2E shows the almost complete blockade of outward TRPM7 currents recorded in 0.4 Ca/0.4 Mg and DVF in the same cell. Unlike with conventional whole-cell recording, the blockade persisted during prolonged recording (up to 9 min, data not shown) suggesting that cytosolic TEA cannot diffuse out of the cell through amphotericin channels.

**FIGURE 3:**
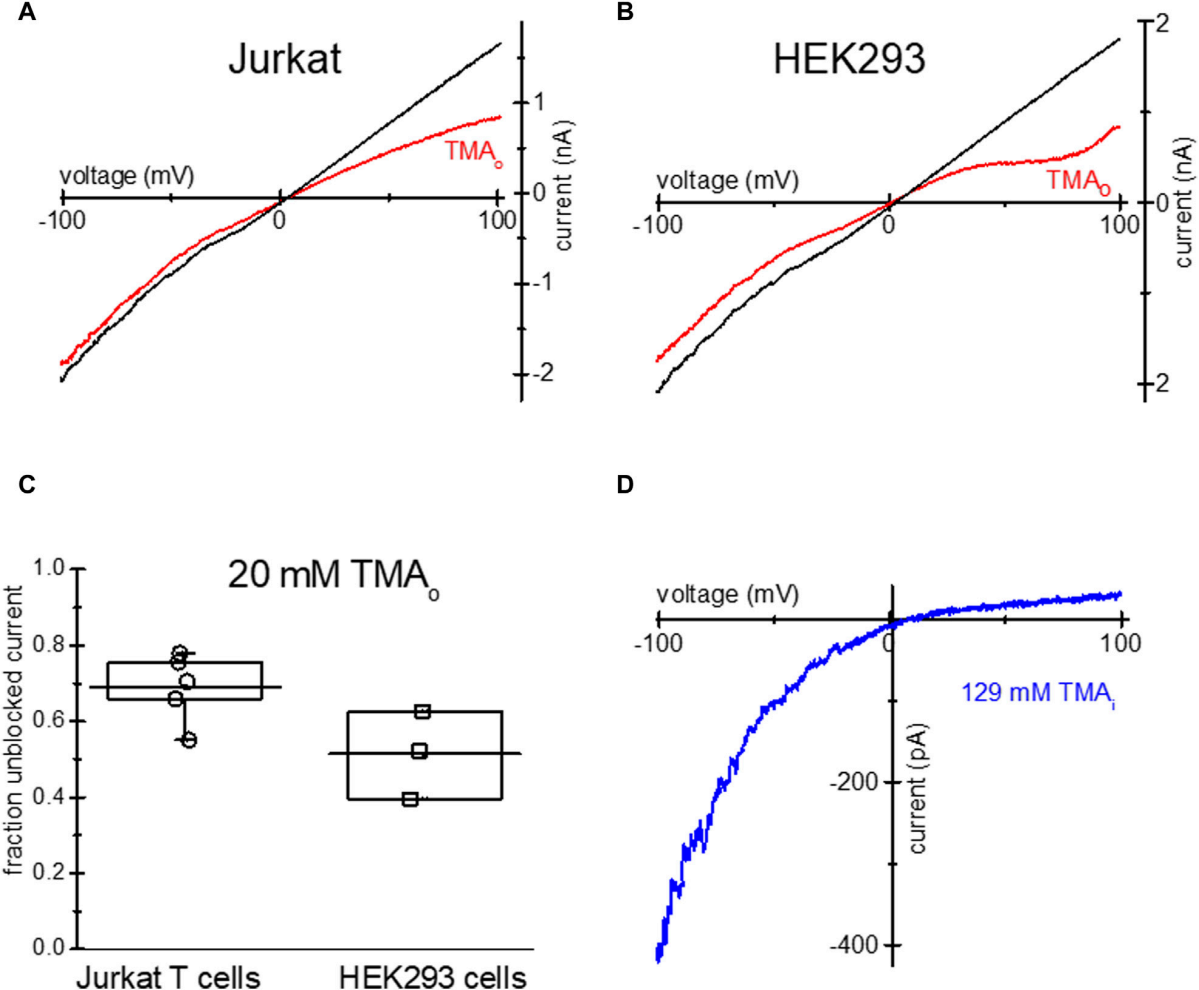
Blockade of TRPM7 monovalent current in TMA-loaded Mg^2+^- depleted Jurkat and HEK293 cells. TRPM7 I-V relations at break in (red) and after 20 mM TMA washout (black) in Jurkat T cell **(A)** and HEK293 cell **(B)** in DVF external solution. **(C)** Summary of TMA uptake experiments, showing the fraction of unblocked monovalent current in both cell lines. **(D)** Blockade of monovalent TRPM7 current by internal 129 mM TMA in a Jurkat T cell. In **(A,B)** the washout trace magnitude was normalized to break-in current magnitude measured at −100 mV.

Similar to TEA, tetramethylammonium (TMA), a closely related quaternary ammonium cation blocks TRPM7 channels at positive membrane potentials (Kozak et al., 2005), we therefore tested if it is taken up by Jurkat and HEK293 cells. Overnight incubation with low Mg^2+^ and 20 mM TMA-Cl resulted in monovalent TRPM7 current blockade in the outward direction (Figures 3A, B). The extent of current blockade measured at 98.67 mV was similar in both human cell lines (Figure 3C). Like TEA (Figure 1), 129 mM TMA in the internal solution blocked most of the outward monovalent current in Jurkat cells (Figure 3D). In conclusion, TMA can be loaded into Jurkat and HEK293, resulting in native TRPM7 channel blockade in intact cells.

### Blockade of heterologously expressed TRPM7 channels by TEA uptake

**FIGURE 4:**
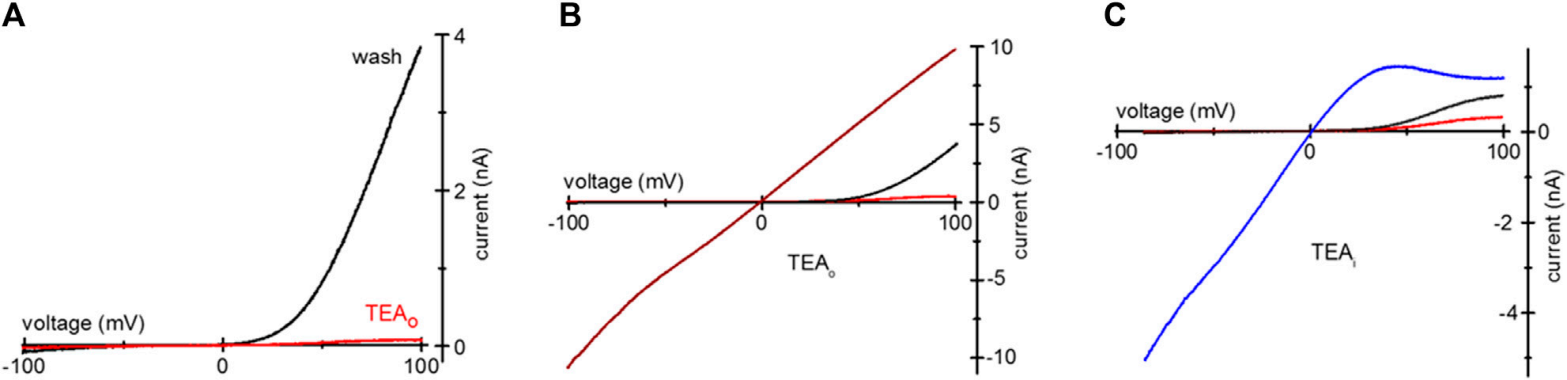
TEA blockade of overexpressed mTRPM7 channels. **(A)** TRPM7 channel current after uptake of 20 mM TEA in a HEK293 cell overexpressing wildtype murine TRPM7. Red and black I-Vs were recorded at break-in and after TEA washout, respectively. **(B)** mTRPM7 currents in a cell loaded with 20 mM TEA recorded at break-in (red), after washout (black) and after removal of divalent cations (wine). **(C)** Blockade of mTRPM7 currents with internal 20 mM TEA. The red and black traces represent break-in and maximal currents. The blue trace represents monovalent current in the same cell, recorded after current had reached maximum in the 0.4 Ca/0.4 Mg solution. In **(C)** the cell was not exposed to external TEA and the command voltage ramps spanned −85 mV–100 mV range.

We tested if TEA loading can be used for inhibition of heterologously expressed TRPM7 channels. HEK293 were transfected with murine TRPM7 cDNA, and TEA was added to normal RPMI medium 1 day after transfection. Unlike the native channels, heterologously expressed TRPM7 shows a substantial basal activity at break-in, without prior Mg^2+^ depletion (Matsushita et al., 2005; Zhelay et al., 2018). Figure 4A shows WT wildtype (WT) TRPM7 channel current blockade in TEA-loaded HEK cells in 0.4 Ca/0.4 Mg. The red trace shows TRPM7 I-V at break-in, and the black trace shows full washout of cytosolic TEA. In Figure 4B, TRPM7 I-Vs were collected immediately after break-in (red), following TEA washout in 0.4 Ca/0.4 Mg (black) and DVF (wine). Inclusion of 20 mM TEA in the internal solution resulted in voltage-dependent block of WT TRPM7 channels at break-in (red), after achieving maximal magnitude (black) and upon switching to DVF (blue) (Figure 4C). As was for endogenous currents (Figures 1, 2), TEA preferentially blocked outward WT TRPM7 currents.

**FIGURE 5:**
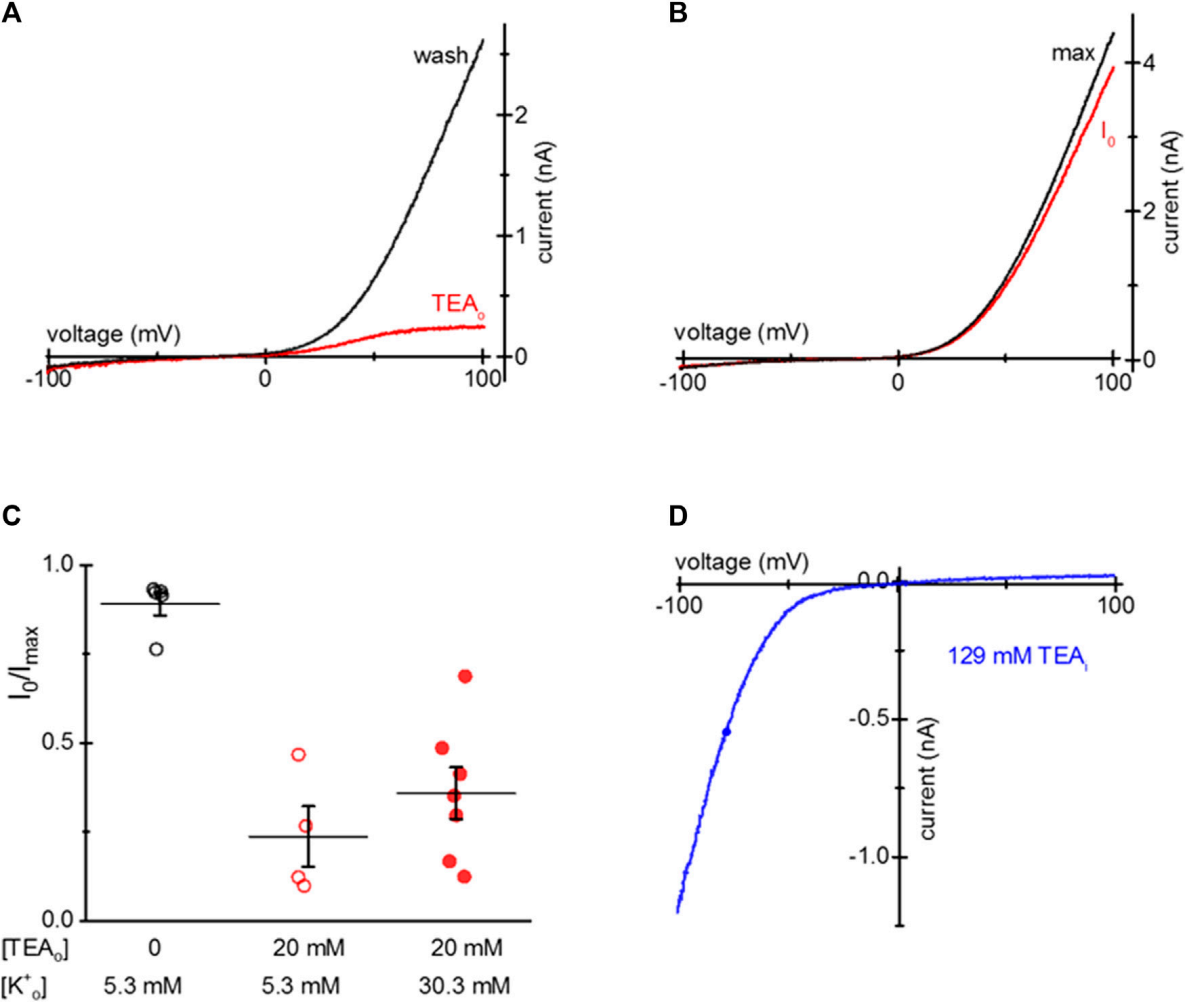
TEA blockade of mTRPM7 S1107R channels. **(A)** TRPM7 S1107R channel current at break-in (red) and after washout (black) in a HEK293 cell after 20 mM TEA uptake. **(B)** S1107R current at break-in and after several minutes of recording in a control HEK293 cell not exposed to TEA. **(C)** Summary of S1107R TRPM7 current block by TEA for HEK cells grown in normal (5.3 mM) and elevated (30.3 mM) [K^+^] containing RPMI in the presence of 20 mM TEA for 1–2 days. The external solution was 0.4 Ca/0.4 Mg. **(D)** Blockade of monovalent S1107R TRPM7 current by internal 129 mM TEA in a HEK293 cell not exposed to external TEA.

S1107R variant forms ion channels that have I-V relations identical to WT TRPM7, but are largely insensitive to cytosolic Mg^2+,^ pH and polyamines (Zhelay et al., 2018). In essence, under physiological ionic conditions S1107R and other substitutions of S1107 make the channel constitutively active (Hofmann et al., 2014; Zhelay et al., 2018). We tested if TEA uptake is effective in blocking S1107R. Figure 5A shows that in HEK cells loaded with 20 mM TEA, outward S1107R current was blocked similar to WT. Figure 5B shows I-V relations recorded at break-in and after maximal current was achieved in a S1107R expressing HEK cell not exposed to TEA.

Our next goal was to test if TEA entry into HEK cells depends on the membrane potential since TEA is a cation. To this end, we supplemented low Mg^2+^ RPMI with 30.3 mM KCl and compared the extent of TEA loading in cells grown in normal (5 mM) and elevated [K^+^]. Figure 5C shows that the extent of TEA blockade was somewhat reduced by depolarizing HEK293 cells but the difference did not reach statistical significance. Inclusion of 129 mM TEA in the pipette blocked most of the outward S1107R current, similar to WT TRPM7 (Figure 5D). These results suggest that TEA block does not depend on Mg^2+^ sensitivity of TRPM7 channels and occurs through a different mechanism, which probably involves pore blockade.

### Blockade of TRPM7 channels by Tris uptake

**FIGURE 6:**
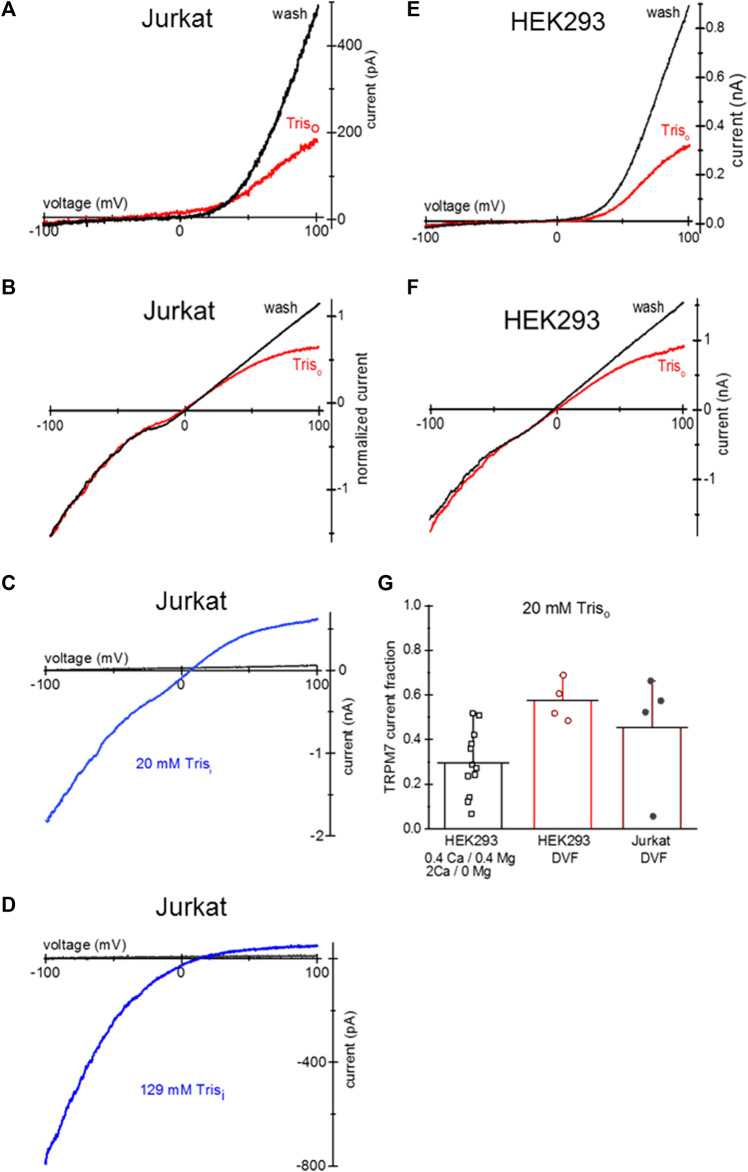
Tris uptake and blockade of TRPM7 channels. **(A,B)** TRPM7 channel current at break-in (red trace) and after washout (black) in a Jurkat T cell loaded with 20 mM Tris-HCl. The external solutions in **(A,B)** were 0.4 Ca/0.4 Mg and DVF, respectively. **(C)** TRPM7 channel monovalent current block by 20 mM Tris added to pipette in a Jurkat T cell. **(D)** TRPM7 channel monovalent current block by internal 129 mM Tris in a Jurkat T cell. In **(C,D)** black traces represent break-in and blue traces maximal current after intracellular Mg^2+^ depletion, respectively. **(E,F)** TRPM7 channel current at break-in (red) and after washout (black) in a HEK293 cell loaded with 20 mM Tris-HCl. The external solutions in **(E,F)** were 0.4 Ca/0.4 Mg and DVF, respectively. **(G)** Summary of the effects of external 20 mM Tris uptake on native TRPM7 in HEK293 cells. Extent of outward current block in 0.4 Ca/0.4 Mg and DVF was calculated as described in Methods. Cells in **(A,B,E,F)** were grown in low Mg^2+^ RPMI for 1–2 days. In **(B)** the current was normalized to −100 mV amplitude.

Tris is a positively charged compound widely used in biochemistry as a pH buffer. Tris-buffered Tyrode’s is used in cell physiology and immunology [e.g., (Okazaki et al., 1975; Hess et al., 1982)]. Similar to TEA, overnight incubation of HEK293 and Jurkat T cells with 20 mM Tris-HCl resulted in the voltage-dependent blockade of endogenous TRPM7 channels (Figure 6). Panels **A** and **B** show TRPM7 current block by 20 mM Tris uptake in Mg^2+^-depleted Jurkat T cells in 0.4 Ca/0.4 Mg and DVF. 20 mM Tris blocked TRPM7 channels in HEK293 cells as well (Figures 6E, F). 20 mM Tris blocked in the outward direction, albeit to a lesser degree than 20 mM TEA: approximately half of outward current was blocked (Figure 6G). Blockade was reversed by diffusion into the patch pipette over several minutes (Figures 6A, B, E, F). Inclusion of 20 and 129 mM Tris in the pipette solution blocked the outward but TRPM7 current in a concentration-dependent manner without affecting the inward current (Figures 6C, D). These experiments demonstrate that Tris is a previously unknown voltage-dependent blocker of TRPM7 channels, and Jurkat and HEK cells can accumulate it after prolonged incubation.

## Discussion

Here we report a simple experimental approach for preferentially blocking outward TRPM7 currents in intact cells by loading with TEA, TMA and Tris. In the presence of external divalent cations, TRPM7 has a characteristic steeply outwardly rectifying I-V relation, reversing near 0 mV. At hyperpolarized membrane potentials, the native TRPM7 channels give rise to a small 10–20 pA inward (negative) current. In the absence of external divalent cations, the inward current is substantially larger and whole-cell I-V is semi-linear with unchanged reversal potential (Kozak et al., 2002; Chokshi et al., 2021). Thus, the ratio of current magnitudes measured at +98 mV and −98 mV, which describes the degree of rectification, is greater than 30 in 0.4 mM Ca/0.4 mM Mg, whereas in DVF it is ∼0.9 (unpublished data). The steep outward rectification arises from preferential block of the inward monovalent current by external Ca^2+^ and Mg^2+^ (Kozak et al., 2002; Kerschbaum et al., 2003). The slight inward rectification of monovalent current is due to smaller single-channel conductance at positive membrane potentials (Chokshi et al., 2012c). Previously, we showed that internal TEA and TMA blocked the outward TRPM7 in human T lymphocytes in DVF solution (Kozak et al., 2005). However, we are not aware of any tools that allow specifically blocking the outward current in intact cells. Here we report that overnight incubation with TEA and TMA block outward TRPM7 current recorded in 0.4 Ca/0.4 Mg bath solution, which mimics RPMI cell culture medium (Figures 1, 2). Blockade by TEA and TMA was fully reversible in whole cell recording. In perforated patch recording, which closely mimics the situation in intact cells, TRPM7 current was blocked in TEA-loaded cells (Figure 2E). In RBL cells, internal 25 mM TEA blocked the monovalent TRPM7 current at positive but not negative membrane potentials (Gwanyanya et al., 2004). TEA blockade of TRPM7 current is reminiscent of voltage dependent blockade by Mg^2+^ and spermine resulting in strong inward rectification of K_ir_ potassium channels such as K_ir_2.1 and K_ir_4.1 (Oliver et al., 2000). Interestingly, for TRPM7 channels intracellular Mg^2+^ and spermine are also inhibitory, but without voltage dependence (Kozak et al., 2002; Kozak et al., 2005; Zhelay et al., 2018).

The majority of studies of TRPM7 cellular function have focused on the inward component of the current, even though it is substantially smaller than the outward component (e.g., Figures 1A, 2A). One of the reasons has been the voltage independence of available inhibitors, which block both inward and outward current components. It has been argued that the small inward TRPM7 current is primarily carried by divalent metal cations Ca^2+^, Mg^2+^, Ba^2+^, Sr^2+^ and others (Monteilh-Zoller et al., 2003; Li et al., 2007; Schmitz et al., 2014; Chubanov et al., 2018; Takahashi et al., 2018). Specifically, TRPM7 channels are thought to mediate cellular Mg^2+^ entry (Monteilh-Zoller et al., 2003; He et al., 2005; Ryazanova et al., 2010; Komiya and Runnels, 2015). We found recently, however, that elimination of inward current by La^3+^ block did not prevent Mg^2+^ uptake, whereas inhibition of *both* inward and outward currents by NS8593 abolished Mg^2+^ entry in the majority of treated Jurkat T cells (Mellott et al., 2020). This suggests that, paradoxically, the outward TRPM7 current may play a role in Mg^2+^ transport into the cell. It should be noted, that a portion of Jurkat T cells exhibited an additional pathway for Mg^2+^ entry even when all TRPM7 channels were blocked.

We report here that intracellular Tris is a voltage-dependent blocker of TRPM7 channels. 20 mM and 129 mM Tris-HCl in the internal solution resulted in a concentration-dependent block of outward TRPM7 current (Figure 6). Similar to TEA and TMA, overnight incubation with 20 mM Tris resulted in its uptake and blockade of outward current in intact cells (Figure 6). Tris did not reduce TRPM7 current magnitude in the inward direction.

Intracellular TEA and TMA block or modify several K^+^ channels including voltage-gated, Ca^2+^-activated K^+^ channels (Hermann and Gorman, 1981; Villarroel et al., 1988; Im and Quandt, 1992; Gonzalez-Perez et al., 2008). Intracellular TEA blocks K_v_1.3 channels which are highly expressed in T cells (Rauer and Grissmer, 1996; Prutting and Grissmer, 2011), thus, uptake of these cations will be expected to block these K^+^ channels also. ATP-gated P2X channels are expressed in T cells and other immune cells (Bohmwald et al., 2021; Vinnenberg et al., 2021) and their properties would be altered upon Tris uptake since this cation is somewhat permeant (Evans et al., 1996).

We did not investigate the identity of pathways for TEA, TMA and Tris entry into Jurkat and HEK293 cells. Maintaining cells in elevated K^+^ (30 mM), which would be expected to depolarize the cells, did not significantly reduce TEA cation loading (Figure 5C). Of note, high extracellular K^+^, reaching 40–50 mM occurs in tumor environment and has a suppressive effect on T lymphocyte function, serving as an “ionic checkpoint” mediating immunosuppression and enabling malignant cells to escape immune attack (Ong et al., 2019). It has been reported that organic cation transporter proteins OCT1 and 2 can serve as pathways for TEA entry in other cell types (Wright et al., 2004; Pelis et al., 2007; Kimura et al., 2009). It would be instructive to determine if HEK293 and Jurkat T cells express the human homologs of OCT proteins.

Future investigation will address several questions of interest: is TEA, TMA, Tris uptake temperature sensitive or can it also occur at room temperature? Does the uptake of these cations depend on TRPM7 channel activity? Based on our findings, entry of TEA is not Mg^2+^ dependent and can occur both in reduced (8 µM) (Figure 1) and normal (0.4 mM) external Mg^2+^ (Figure 4). This makes it unlikely that TRPM7 participates in the uptake of these cations. TEA and TMA uptake may occur through other channels. For example, aquaporin 1 (AQP1) water channels also conduct cations K^+^, Cs^+^, Na^+^ and TEA, likely through the central pore of the AQP1 tetramer (Boassa et al., 2006; Yu et al., 2006). Pilot data showed that incubating cells in presence of TEA, TMA and Tris for up to 2 days, was not visibly toxic, and can therefore be used for long-term experiments aiming to dissect the physiological roles of TRPM7 channels.

## Data Availability

The raw data supporting the conclusion of this article will be made available by the authors, without undue reservation.
